# Protocol of global incidence and progression of age-related macular degeneration: A systematic review

**DOI:** 10.1097/MD.0000000000014645

**Published:** 2019-03-08

**Authors:** Shan Zhao, Xiaowen Lan, Jingyang Wu, Song Yue, Han Zhang, Qiang Wu, Guisen Zhang, Lei Liu

**Affiliations:** a Department of Rheumatology and Immunology, First Affiliated Hospital, China Medical University, Shenyang; b Datong Chaoju Eye Hospital, Datong; c Department of Ophthalmology, First Affiliated Hospital, China Medical University, Shenyang; d Hohhot Chaoju Eye Hospital, Hohhot, People's Republic of China.

**Keywords:** age-related macular degeneration, burden, incidence, meta-analysis, progression

## Abstract

**Background:**

There have been many reports on the prevalence and incidence of age-related macular degeneration (AMD), and there are some systematic reviews reporting on the pooled prevalence of AMD. However, there is no systematic review of incidence or progression of AMD worldwide. Given the few evidences regarding the pooled incidence or progression of AMD, we performed this meta-analysis protocol to investigate the global incidence or progression of AMD. In addition, we will investigate the risk factors for AMD incidence or progression using meta-analysis.

**Methods:**

Four English databases (PubMed, EMBASE, Cochrane Library, and Web of Science) and four Chinese databases (CMB, CNKI, VIP, and Wanfang database) will be searched to identify relevant studies. The primary outcome of this meta-analysis is the incidence or progression of AMD. The second outcome of this meta-analysis is risk factors for the incidence or progression of AMD. Meta-analysis was performed to calculate the pooled incidence or progression rate and 95% confidence interval of AMD. Pooled risk ratios of risk factors (age, gender, smoking, and hypertension) for AMD incidence or progression were computed as the Mantel–Haenszel-weighted average of the risk ratios for all included studies. Sensitivity analysis, subgroup analysis, quality assessment, and publication bias analysis will be performed to ensure the reliability of our findings.

**Results:**

This study will provide a current evidence of global pooled incidence or progression of AMD. Further, current study will provide evidence-based risk factors for AMD incidence or progression. Moreover, our study will project the incident number of people with AMD from 2030 to 2050.

**Conclusion:**

This systematic review and meta-analysis will provide evidence to develop major public health strategies for preventing AMD. Ethics and dissemination: ethical approval is not required because our systematic review and meta-analysis will be based on published data without interventions on patients. The findings of this study will be published in a peer-reviewed journal.

## 1 Introduction

With the increasing numbers of people living longer, the number of people with age-related diseases is rising worldwide. Age-related macular degeneration (AMD) is a common age-related disease which is also a leading cause of visual impairment and severe vision loss.^[1]^ To date, prevalence of AMD is likely to increase due to exponential population ageing.^[2]^

There have been some studies on the incidence and prevalence of AMD, with systemic reviews summarizing global estimates of its prevalence across regions.^[2]^ Moreover, there is 1 report on late-AMD incidence among American Whites^[3]^ which showed annual incidence of late AMD was 3.5 per 1000 aged ≥50 years. In particular, there is no systematic review of incidence or progression of AMD worldwide and little is known about the incidence and progression of early stage of AMD and the disease in other parts of population apart from American Whites. Furthermore, interpreting incidence estimates from different studies on the incidence of AMD is challenging because of significant variation in its estimates between ethnicities and regions, due to differences in study setting, method of ascertainment of AMD, and follow-up time trends. Robust data on incidence and progression of AMD are important for development of major public health strategies to prevent this disease.

To address this gap, we conducted a systematic review and meta-analysis to estimate the global incidence or progression of AMD, and to describe variations by ethnicity, region, study characteristics, and follow-up time period in which the studies were conducted.

## 2 Methods

### 2.1 Design and reporting

This systematic review will be designed according to the Preferred Reporting Items for Systematic Review and Meta-Analysis (PRISMA) guidelines.^[4]^ For current protocol, the PRISMA statement for Protocols (PRISMA-P) was used for its description (Table 1).^[5]^ This systematic review is registered in the PROSPERO International Prospective Register of systematic reviews with number CRD42019118832.

**Table 1:**
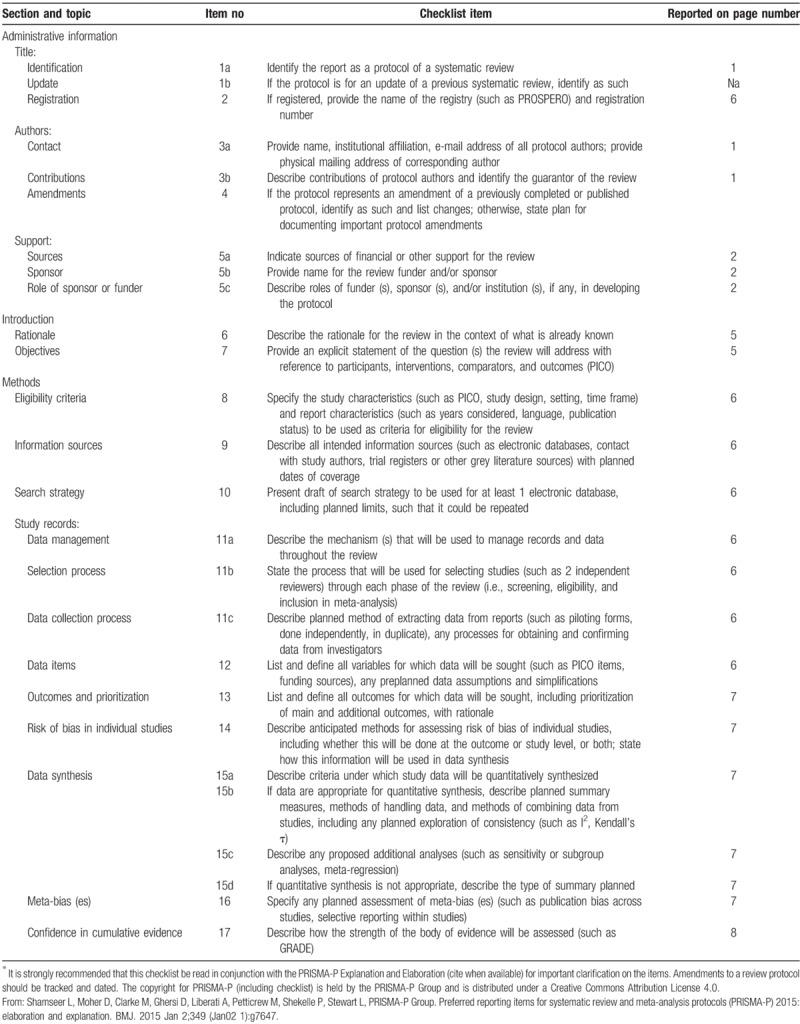
PRISMA-P (Preferred Reporting Items for Systematic review and Meta-Analysis Protocols) 2015 checklist: recommended items to address in a systematic review protocol^∗^.

### 2.2 Data source and search strategy

Published primary studies will be gathered using four English databases (PubMed, EMBASE, Cochrane Library, and Web of Science) and four Chinese databases (CMB, CNKI, VIP, and Wanfang database). References of the relevant articles will be searched by hand. Moreover, for article which is difficulty accessing sufficient data or full text, its corresponding author will be contacted by e-mail. The key search terms will be “incidence,” “development,” “associated factors,” “progression,”’ and “age-related macular degeneration.” Using all these terms, relevant topics will be searched through ‘All fields’ using the connecting ‘AND’ and ‘OR’ as appropriate.

### 2.3 Inclusion criteria

Type of studies: prospective or retrospective cohort studies.

Type of participants: population over 40 years old.

Type of outcome: incidence or progression (or studies giving enough data to compute these estimates if not directly calculated) of AMD.

Language: English or Chinese.

### 2.4 Exclusion criteria

Type of studies: case–control studies, cross-sectional studies, case reports, case series, letters, reviews, and editorials.

Duplicate reports.

### 2.5 Selection of studies for inclusion in the review

Articles will be identified by 1 clinical scientist and reviewed by another senior clinical scientist. Data will be evaluated by a statistician, and consensually retain studies to be included. Disagreements when existing will be solved by discussion.

### 2.6 Data extraction and management

Data will be extracted using a designed form. Two reviewers will independently extract data. The domains included study setting (title, follow-up time, design, and region), study population (age, gender, and ethnicity), method of ascertainment of AMD, and information on severity level of AMD.

### 2.7 Appraisal of methodological quality of included studies and risk of bias

Methodological quality for included studies will be evaluated using the 10-item rating scale (Table 2).^[6]^ Each item will be assigned a score of 1 (yes) or 0 (no), and each score will be summed across items to generate an overall study quality score. Included studies will be defined into 3 levels according to overall score as follows: low risk of bias (8–10), moderate risk (6–7), and high risk (0–5).

**Table 2:**
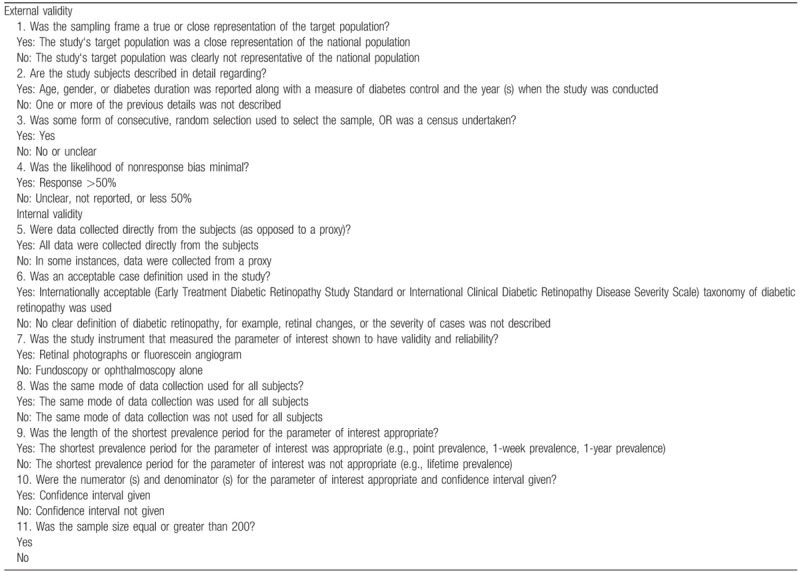
Quality assessment.

### 2.8 Data synthesis

Incidence of AMD was calculated as cumulative incidence including both early AMD and late AMD and any-AMD. According to included studies varied in time of follow-up, we calculated annual incidence of AMD using the formula −ln (1 − S)/t, where S is the proportion of new AMD cases over t years and t is the time of follow-up.^[7]^ Similar to the incidence of AMD, we will calculate the cumulative progression and the annual progression estimates of AMD. We will perform subgroup analysis on the incidence of AMD by study region, population age and gender, follow-up duration, and method of ascertainment of AMD. We will also assess the effect of major risk factors for AMD incidence including age, gender, ethnicity, smoking, and others wherever data were available.

### 2.9 Assessment of reporting biases

The presence of publication and selective reporting bias will be assessed using symmetry of funnel plots and Egger's test.^[8]^ Asymmetry of the funnel plot or a *P* value of Egger's regression test less than 0.05 will be considered indicative of significant publication bias.

### 2.10 Ethics and dissemination

Ethics approval is not required as this is a systematic review and meta-analysis using published data. We will report our findings of this systematic review and meta-analysis in a peer-reviewed journal in future.

## 3 Discussion

The burden of age-related diseases is increasing in China, as a common age-related eye disease, AMD is becoming a common cause of visual impairment and blindness in elder population. In this comprehensive systematic review and meta-analysis, we will include cohort studies regarding the incidence or progression of AMD worldwide. Moreover, this systematic review and meta-analysis will provide summarized data to establish global incidence or progression estimates and its risk factors. Furthermore, current systematic review and meta-analysis will project the number of people with AMD from 2030 to 2050 which will be a useful guide for public health strategies to control AMD.

## Author contributions

**Author contributions:** L.L. developed the study protocol. S.Z. and X.W.L. developed the search strategy. J.Y.W. and S.Y. will scan the included studies. H.Z. and G.S.Z. extract the data and assess the risk of bias. L.L. will act as an arbiter if there is any disagreement in this study. L.S. and Q.W. will perform data analysis. All authors will contribute to data interpretation. S.Z. and L.L. drafted and revised the manuscript. All authors have read and approved the final version of the manuscript.

**Data curation:** Xiaowen Lan.

**Formal analysis:** Jingyang Wu, Qiang Wu.

**Investigation:** Jingyang Wu.

**Methodology:** Shan Zhao, Song Yue, Guisen Zhang.

**Project administration:** Han Zhang.

**Software:** Song Yue.

**Supervision:** Lei Liu.

**Validation:** Lei Liu.

**Writing – original draft:** Shan Zhao, Xiaowen Lan, Han Zhang, Qiang Wu, Guisen Zhang, Lei Liu.

**Writing – review & editing:** Shan Zhao, Guisen Zhang, Lei Liu.
